# Glass-Ceramic Synthesis of Cr-Substituted Strontium Hexaferrite Nanoparticles with Enhanced Coercivity

**DOI:** 10.3390/nano11040924

**Published:** 2021-04-05

**Authors:** Lev A. Trusov, Anastasia E. Sleptsova, Jingtong Duan, Evgeny A. Gorbachev, Ekaterina S. Kozlyakova, Evgeny O. Anokhin, Artem A. Eliseev, Maxim A. Karpov, Alexander V. Vasiliev, Oleg A. Brylev, Pavel E. Kazin

**Affiliations:** 1 Faculty of Materials Science, MSU-BIT University, Shenzhen 517182, China; djt2010bj@126.com (J.D.); ev.a.gorbachev@gmail.com (E.A.G.); brylev@inorg.chem.msu.ru (O.A.B.); 2 Faculty of Chemistry, Lomonosov Moscow State University, 119991 Moscow, Russia; anokhin.evgeny@gmail.com (E.O.A.); artem.a.eliseev@gmail.com (A.A.E.); maksim.carpow2010@yandex.ru (M.A.K.); a.vasiliev@inorg.chem.msu.ru (A.V.V.); kazin@inorg.chem.msu.ru (P.E.K.); 3 Faculty of Materials Science, Lomonosov Moscow State University, 119991 Moscow, Russia; sleptsovaanastasia@gmail.com; 4 Faculty of Physics, Lomonosov Moscow State University, 119991 Moscow, Russia; evenuel1@gmail.com; 5 Laboratory of Functional Quantum Materials, National University of Science and Technology “MISiS”, 119049 Moscow, Russia

**Keywords:** strontium hexaferrite, hard ferrites, hard magnetic materials, magnetic nanoparticles, nanomagnets

## Abstract

Magnetically hard ferrites attract considerable interest due to their ability to maintain a high coercivity of nanosized particles and therefore show promising applications as nanomagnets ranging from magnetic recording to biomedicine. Herein, we report an approach to prepare nonsintered single-domain nanoparticles of chromium-substituted hexaferrite via crystallization of glass in the system SrO–Fe_2_O_3_–Cr_2_O_3_–B_2_O_3_. We have observed a formation of plate-like hexaferrite nanoparticles with diameters changing from 20 to 190 nm depending on the annealing temperature. We demonstrated that chromium substitution led to a significant improvement of the coercivity, which varied from 334 to 732 kA m^−1^ for the smallest and the largest particles, respectively. The results provide a new strategy for producing high-coercivity ferrite nanomagnets.

## 1. Introduction

Hard magnetic hexaferrites MFe_12_O_19_ (M = Ba, Sr) are well known and widely used materials in the production of ceramic permanent magnets [1,2,3]. However, they are also very promising for various applications in nanotechnology. Due to high magnetocrystalline anisotropy, even very small hexaferrite nanoparticles do not turn to a superparamagnetic state and remain hard-magnetic, preserving a high coercivity and permanent magnetization. Moreover, unlike most metallic magnetic nanoparticles (e.g., FePt, Co, CoPt, NiFe), hexaferrites are thermally stable, chemically robust, and biocompatible. Thus, the hexaferrite nanoparticles are a reasonable material when nanomagnets are required for diverse purposes. For example, hexaferrites are attractive for high-density magnetic recording tape media [1,4,5] and low-frequency magnetic hyperthermia [6], as well as for magnetic colloids of hard magnetic particles, which are hugely different from traditional magnetite-based ferrofluids and demonstrate several unique properties [7,8,9]. Certainly, the colloidal hexafeFrrite nanoparticles are building blocks for the creation of magnetic nanostructures [10,11], nanocomposites [12,13], and various nanomaterials [14,15,16,17,18].

Though there are plenty of techniques for obtaining hard magnetic ferrites, such as ceramic, sol-gel, chemical coprecipitation syntheses, etc. [1], most of them include a high-temperature treatment step that leads to particle agglomeration and sintering. As a result, the products are not suitable for application in nanotechnology. Glass crystallization is a powerful method for the production of composite materials, where hexaferrite nanoparticles are separated from each other by a nonmagnetic matrix [19,20]. The most convenient are glasses in the SrO–Fe_2_O_3_–B_2_O_3_ system, since the matrix is formed by borates, which are easily soluble in weakly acidic solutions. Consequently, the pure hexaferrite phase can be extracted in the form of fine powders or stable colloids [8,10].

The most important functional property of the hard hexaferrites is their coercivity, which reaches a maximum at about 520 kA m^−1^ (6500 Oe) for single-domain particles with diameters below 500 nm [1,21]. However, below the particle diameter of approximately 100 nm, the coercivity is highly affected by size and shape effects, which results in its significant reduction. The particles smaller than 10 nm become superparamagnetic and lose their coercivity. Typical reported colloidal hexaferrite nanoparticles with diameters of 10–100 nm display coercivity values between zero and 360 kA m^−1^ (4500 Oe) for the largest particles [7,15,22,23,24]. A common way to increase the coercivity of the hexaferrites is the partial substitution of iron ions with aluminum [1,3,21,25,26,27], e.g., this can result in a coercivity of submicron single-domain particles of up to 3180 kA m^−1^ (40 kOe) [25]. However, the aluminum substitution requires high annealing temperatures above 1000 °C, which makes the preparation of nanoparticles quite challenging. The crystallization of glasses in the system SrO–Fe_2_O_3_–Al_2_O_3_–B_2_O_3_ could be a solution [28,29]; however, the high substitution degrees are still not achieved, probably due to the high glass-forming ability of aluminum oxide in the presence of strontium oxide [30]. Nevertheless, this approach allowed one to produce nonsintered submicron hexaferrite particles with coercivities over 800 kA m^−1^ (10 kOe) [29] and colloidal nanoparticles with a coercivity of up to 445 kA m^−1^ (5600 Oe) [7]. Chromium substitution should have a similar coercivity increase effect [1,3,21,31]; however, the preparation of nonsintered submicron or nanosized particles of Cr-substituted hexaferrite has not been described so far.

Here we report for the first time a synthesis of chromium-substituted hexaferrite by the crystallization of glass in the system SrO–Fe_2_O_3_–Cr_2_O_3_–B_2_O_3_ and discuss the morphology of the particles and their magnetic properties in relation to the annealing temperature.

## 2. Materials and Methods

The glass of the initial composition SrFe_8_Cr_4_O_19_–12Sr_2_B_2_O_5_ was prepared by rapid melt quenching. For that purpose, the stochiometric mixture of the starting reagents (SrCO_3_, Fe_2_O_3_, H_3_BO_3_, and Cr_2_O_3_, high purity grade, Aldrich, Saint Louis, MO, USA) was homogenized into a 10 g batch and fired at 900 °C for 2 h; then, the batch was ground and melted in a platinum crucible at 1350 °C. After a 1 h exposure, the melt was quenched between two rotating steel rollers to form glassy flakes. The glass ceramics were formed by isothermal heat treatment of the glass at annealing temperatures (*T*_ann_) of 600–900 °C during 2 h. Then, the samples were treated with 3% hydrochloric acid to dissolve the borate matrix and to leach the magnetic particles, which were separated from the solution by centrifugation, thoroughly washed with distilled water, and dried.

A differential thermal analysis (DTA) of the glass was carried out in air on a Netzsch (Selb, Germany) STA 409 PC Luxx instrument with a heating rate of 5 °C/min. The Curie temperatures were determined by thermogravimetric analysis with a heating rate of 10 °C/min (a PerkinElmer (Waltham, MA, USA) Pyris Diamond TG/DTA instrument) in the magnetic field of an NdFeB magnet. Powder diffraction studies (XRD) of glass-ceramics were performed using a Rigaku (Tokyo, Japan) D/Max-2500 diffractometer (CuKα radiation), and hexaferrite powders were measured on a Stoe (Darmstadt, Germany) Stadi-P (MoKα_1_ radiation). The Rietveld analysis of the XRD patterns was carried out using the MAUD program (version 2.94) [32]. The microstructure was examined using a scanning electron microscope Zeiss (Oberkochen, Germany) LEO Supra 50 VP (SEM) equipped with an Oxford Instruments (Oxfordshire, UK) Energy+ detector for Energy-dispersive X-ray spectroscopy (EDX analysis) and a transmission electron microscope Carl Zeiss (Oberkochen, Germany) Libra 200MC (TEM). A chemical analysis was also performed by the inductively coupled plasma mass spectrometry method (ICP-MS) using a PerkinElmer (Waltham, MA, USA) Elan DRC II instrument. The magnetic hysteresis loops were recorded at room temperature using a Cryogenic (London, UK) SQUID magnetometer S700 in fields of up to 3 T.

## 3. Results and Discussion

**Figure 1:**
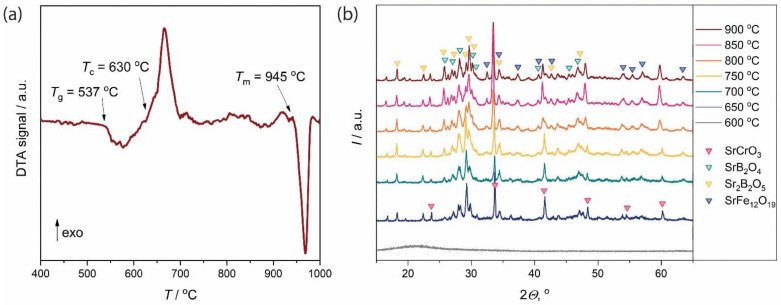
(**a**) DTA curve of the SrFe_8_Cr_4_O_19_–12Sr_2_B_2_O_5_ glass; (**b**) XRD patterns of the glass-ceramics. The annealing temperatures and observed phases are denoted on the right.

According to the XRD analysis, the melt-quenched sample contained no crystalline phases. It showed a paramagnetic response that indicated a lack of ferromagnetic phases, e.g., magnetic iron oxides or hexaferrite. The SEM analysis of the polished sample confirmed its homogeneity down to the nanometer scale. Thus, the rapid melt quenching technique allows for the production of an amorphous glass precursor. The DTA measurement (Figure 1a) revealed a glass transition temperature of 537 °C and an exothermal effect corresponding to glass devitrification, which started at 630 °C and peaked at 665 °C. At about 945 °C, the melting of the sample began. The observed characteristic temperatures were close to those previously reported for the SrFe_12_O_19_–12Sr_2_B_2_O_5_ glass [33].

**Figure 2:**
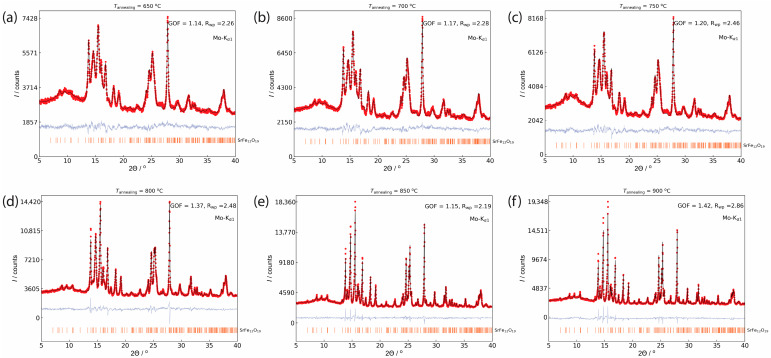
XRD patterns and Rietveld analyses of the extracted hexaferrite powders obtained at different annealing temperatures: (**a**) 650 °C; (**b**) 700 °C; (**c**) 750 °C; (**d**) 800 °C; (**e**) 850 °C; (**f**) 900 °C. Black lines, red lines, and light blue lines are the observed and calculated patterns, and their differences, respectively. The orange bars correspond to the peak position of the hexaferrite phase (ICDD PDF 84-1531).

The powder x-ray diffraction patterns of the glass ceramics obtained by isothermal heat treatment at 600–900 °C are shown in Figure 1b. The sample obtained at 600 °C remained amorphous, and the crystalline phases appeared after annealing at 650 °C in accordance with the exothermal peak on the DTA curve. Due to the complex glass composition and presence of nanosized particles, the accurate phase analysis is quite difficult. The main crystalline phases in the glass-ceramics are strontium borates SrB_2_O_4_ (ICDD PDF 84-2175) and Sr_2_B_2_O_5_ (ICDD PDF 73-1930), as well as SrCrO_3_ (ICDD PDF 20-1192) most likely substituted by iron. The peaks of the hexaferrite phase SrFe_12_O_19_ (ICDD PDF 84-1531) become visible on the XRD patterns only above 800 °C. Nevertheless, the treatment of the glass-ceramics annealed above 650 °C with 3% HCl resulted in the complete dissolution of the nonmagnetic phases and the separation of pure single-phase hexaferrite powders (Figure 2). The unit cell parameters (Table 1) are slightly reduced from those of SrFe_12_O_19_ (*a* = 5.885 and *c* = 23.05 Å [34]) due to the smaller ionic radius of the Cr^3+^ ion (*r*^VI^ = 0.615 Å) in comparison with the Fe^3+^ ion (*r*^VI^ = 0.645 Å) [35]. The lattice parameters in all the samples are very close, indicating only a small variation of the chromium substitution ratio in the samples. However, the hexaferrite composition is hard to estimate from the lattice parameters, e.g., by Vegard’s law, because of their weak dependence on the chromium content and the lack of reliable reported data in the literature.

The diffraction lines are considerably broadened for the annealing temperatures up to 800 °C, which indicates nanoscale particle sizes in these samples. Furthermore, the (*h k 0*) diffraction lines are noticeably narrower than the lines with *l*-indices, which corresponds to a strong anisotropy of the particles with a smaller dimension along the crystallographic *c*-axis, i.e., to a plate-like particle shape. The nanoparticle dimensions estimated by the Rietveld refinement of the profiles are summarized in Table 1 (*d* and *h* correspond to the diameter and thickness of the nanoplates). The rise of the annealing temperatures from 650 °C to 750 °C leads to a slight increase in the mean particle diameter from 19.9 nm to 24.2 nm. The following elevation of the annealing temperature to 800 °C results in a pronounced particle enlargement up to a mean diameter of 61.7 nm, while the *d*/*h* ratio remains about 5.

**Figure 3:**
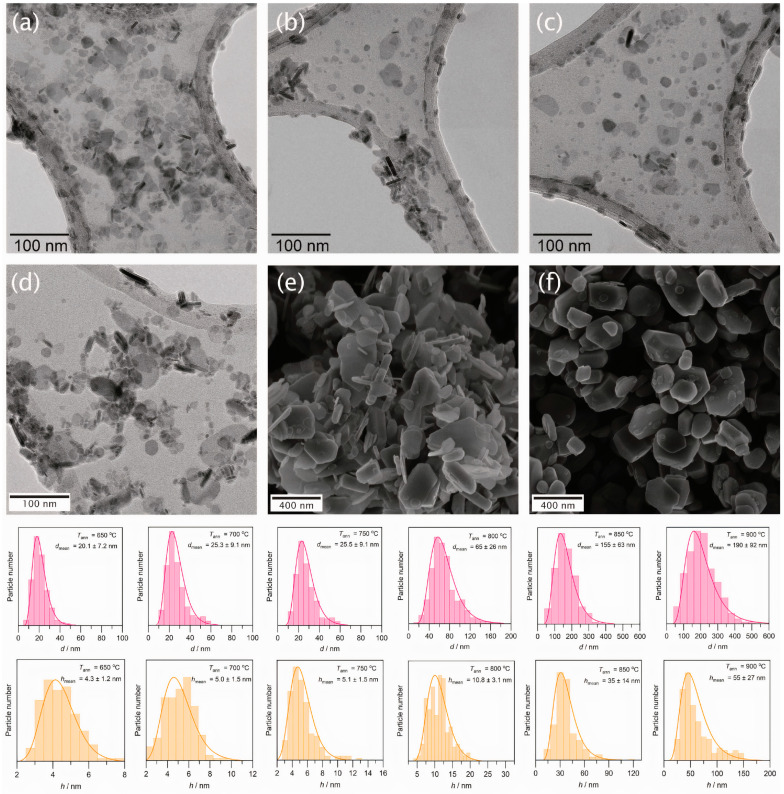
Electron microscopy images of the extracted hexaferrite powders obtained at different annealing temperatures: (**a**) 650 °C; (**b**) 700 °C; (**c**) 750 °C; (**d**) 800 °C; (**e**) 850 °C; (**f**) 900 °C. The corresponding size distributions are shown below.

The electron microscopy confirmed the results of the Rietveld refinement for hexaferrite nanoparticles obtained at 650–800 °C (Figure 3a–d). The particles reveal a shape of irregular plates with diameters below 100 nm. At higher annealing temperatures the unfreezing of the diffusion processes within the matrix of the glass-ceramic led to a recrystallization by the Ostwald ripening. At *T*_ann_ = 850 °C, the hexaferrite powder contains both small nanoplates and submicron particles with well-defined facets (Figure 3e), and at *T*_ann_ = 900 °C the sample consists mainly of large, faceted particles with a mean diameter of 190 nm (Figure 3f). Consequently, the hexaferrite particles in all samples should be in the single-domain state, since their sizes are significantly lower than the critical diameter of a single-domain hexaferrite particle (500 nm for the lower estimate [21]).

The results of the chemical analysis by ICP-MS are shown in Table 2. The chromium content in SrFe_12-x_Cr_x_O_19_ varies from *x* = 1.72 to *x* = 2.32, with a maximum for the sample annealed at 750 °C. It is worth noting that the chromium content increases with the temperature in the samples where nanoparticles are formed (*T*_ann_ = 650–750 °C), while at higher annealing temperatures it decreases. The reason may be the formation of chromium-depleted secondary hexaferrite during the observed recrystallization process. The measured Curie temperatures are lower than those of pure SrFe_12_O_19_ (740 K [21]), which also indicates the Cr substitution. However, the Curie temperatures of the nanoparticles are additionally reduced, probably due to size effects.

**Figure 4:**
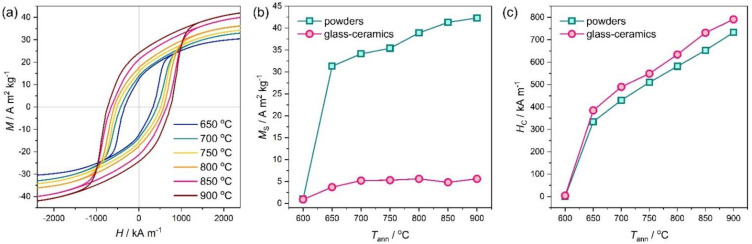
(**a**) Hysteresis loops of the extracted hexaferrite powders; (**b**) The saturation magnetization of the glass-ceramics and extracted hexaferrite powders; (**c**) The coercivity of the glass-ceramics and extracted hexaferrite powders.

The magnetic hysteresis loops of the extracted hexaferrite particles and corresponding magnetic properties (the saturation magnetization *M*_S_ and the coercivity *H*_C_) dependent of the annealing temperature are shown in Figure 4. Furthermore, the magnetic characteristics are summarized in Table 1. The sample annealed at 600 °C dissolved almost completely, and the remaining powder displayed paramagnetic behavior. Starting from the annealing temperature of 650 °C, the samples possess pronounced hysteresis loops typical for randomly oriented single-domain Stoner–Wohlfarth particles with uniaxial magnetocrystalline anisotropy (*M*_R_/*M*_S_ ≈ 0.5) [36]. This conforms to the results of the XRD analysis and indicates the formation of a magnetically hard hexaferrite phase after annealing at 650 °C and above. However, the saturation magnetization of the glass-ceramics is significantly lower than is expected from the theoretical SrFe_8_Cr_4_O_19_ content (about 24 wt. %). Comparing the saturation magnetization of the glass-ceramics with the magnetization of single-phase hexaferrite powders, we obtain a hexaferrite content of about 12 wt.%, which lower than expected by a factor of two. This agrees with the phase analysis, which showed a large amount of SrCrO_3_ in the glass-ceramics.

The coercivity of the powder obtained at 650 °C is 334 kA m^−1^ (4200 Oe) and then gradually rises when increasing the annealing temperature and, consequently, the particle size. At *T*_ann_ = 900 °C, the coercivity reaches 732 kA m^−1^ (9200 Oe). The coercivity of nanopowders is strongly affected by particle size effects. The hexaferrite particles with a diameter below approximately 10 nm are supposed to be superparamagnetic and show no magnetic hysteresis (i.e., zero coercivity) [22], and exceeding this size leads to the appearance of the coercivity and its increase with a diameter of up to about 40–60 nm (estimation for a spherical particle), when the Stoner–Wohlfarth model of coherent rotation becomes valid [1,21]. Moreover, at higher temperatures, thicker particles are formed (Table 1), which have lower demagnetization factors and therefore increased magnetic anisotropy [37]. Additionally, larger particles possess fewer structural and surface defects, which usually contribute to a decrease in the magnetic hardness. All these factors cause the observed rise in the coercivity. On the other hand, the changes in the chromium content in the samples are not great, so they have an insignificant effect on the variation of the magnetic properties. The saturation magnetization of the nanopowders is also reduced due to rising surface effects and the non-collinear spin orientation [1,23].

The coercivity of the glass-ceramics is even higher than that of the powders by 40–80 kA m^−1^ (500–1000 Oe) and runs up to 795 kA m^−1^ (10,000 Oe) at *T*_ann_ = 900 °C. The coercivity reduction after extraction may be explained by the effect of the particle aggregation. The particles in the glass-ceramics are in general separated by nonmagnetic phases, while in the powder they are in close contact and therefore demagnetize each other by magnetic dipole–dipole interactions. Furthermore, the influence of the treatment by hydrochloric acid cannot be excluded because it may lead to a smaller particle size or change the surface of the particles. The same observations were previously reported, e.g., for pure as well as aluminum substituted hexaferrite nanoparticles [29,38].

To determine the effect of chromium substitution, it is worth comparing the obtained magnetic properties (Table 1) with those reported previously for pure hexaferrite with similar particle dimensions. For example, in the work [38], the particles of SrFe_12_O_19_ were produced by the same glass crystallization method and the particle sizes varied from 20×5 nm^2^ (*M*_S_ = 53.7 A m^2^ kg^−1^, *H*_C_ = 175 kA m^−1^) to 270×100 nm^2^ (*M*_S_ = 70.0 A m^2^ kg^−1^, *H*_C_ = 454 kA m^−1^). As we can see, the saturation magnetization of the Cr-substituted hexaferrite is reduced, which is explained by the chromium preference to first occupy the 2*a* and 12*k* sites with uncompensated spins [21,39]. At the same time, the coercivity is increased by 90% for the smallest nanoparticles and by 60% for the submicron ones. The coercivity of the nanoparticles is also higher than that obtained by most of the other methods [15,24,37,40]. As compared with Al-substituted hexaferrites prepared by glass crystallization [7,29], Cr-substituted powders possess very similar properties for larger particles; however, they allow for the production of much smaller nanoparticles with enhanced coercivity. Although the smallest ferrite nanomagnet made of ε-Fe_2_O_3_ reveals higher coercive forces (e.g., 270 kA m^−1^ and 660 kA m^−1^ for spherical 8.2-nm and 10.5-nm particles, respectively) [41], Cr-substituted hexaferrite has a magnetization that is at least two times higher and features a plate-like particle shape, which may attract additional attention, for example due to the unique magneto-optical properties [9] or the ability to self-organize [10]. Moreover, the used glass-ceramic technique provides room for the future optimization of particle size and morphology as well as an increase in the chromium content, which should lead to an improvement in the coercivity.

Surprisingly, the chromium substitution in hexaferrites is quite rarely reported and is therefore not well investigated. This may be due to early studies, which revealed the reduction of the anisotropy field *H*_A_ with an increasing chromium content in a hexaferrite structure [21,42]. This should result in low coercivity even for single-domain particles and, hence, a depreciation of scientific interest. On the contrary, our findings unambiguously show that the incorporation of chromium ions into the hexaferrite structure causes a considerable increase in coercivity, which is especially important for nanoparticles.

## 4. Conclusions

In summary, we have obtained Cr-substituted single-domain hexaferrite particles via a glass-ceramic method. The incorporation of chromium ions into a hexaferrite structure results in a considerable increase in the coercivity and, to date, the highest reported size/coercivity ratio for hexaferrite nanoparticles. In particular, plate-like particles with dimensions of 20 × 4, 25 × 5, and 65 × 11 nm^2^ possess a coercivity of 334, 509, and 581 kA m^−1^, respectively. The coercivity of larger 190 × 55 nm^2^ particles reaches 732 kA m^−1^. The synthesis method provides nonsintered particles, which can be used in various fields where nanomagnets are needed, e.g., in durable magnetic recording media, electromagnetic wave shielding, magnetic force microscopy tips, ferrofluids with a magnetically adjustable refractive index, magneto-mechanical microsystems, and magnetic self-assembled nanostructures.

## Figures and Tables

**Table 1:** Properties of the hexaferrite particles extracted from glass-ceramics.

*T*_ann_ °C	Lattice parameters		Mean size^1^		Mean size^2^		*M*_S_ A m^2^ kg^−1^	*H*_C_ kA m^−1^	*T*_C_
	*a*, Å	*c*, Å	*d*, nm	*h*, nm	*d*, nm	*h*, nm			K
650	5.8749(4)	23.037(3)	19.9	3.8	20.1	4.3	31.3	334	622
700	5.8747(3)	23.014(3)	23.6	4.8	25.3	5.0	34.1	430	637
750	5.8744(4)	22.998(3)	24.2	4.8	25.5	5.1	35.4	509	653
800	5.8745(2)	22.990(1)	61.7	12.0	65.0	10.8	38.9	581	661
850	5.8745(1)	22.9901(5)	/		155	35	41.3	653	658
900	5.8746(1)	22.9908(5)	/		190	55	42.3	732	658

^1^ Particle dimensions estimated from the full-profile analysis of x-ray diffraction patterns by the Rietveld method (*d* – diameter, *h* – thickness of the plate-like particle).^2^ Mean particle dimensions obtained by approximating TEM (650–800 °C) and SEM (850 and 900 °C) histograms (Figure 3) with a lognormal distribution function (*d*—diameter, *h*—thickness of the plate-like particle).

**Table 2:** Chemical analysis of hexaferrite particles’ composition ^1^.

*T*_ann_ °C	ICP-MS, at. Ratio		
	Sr	Fe	Cr
650	0.86	9.88	2.12
700	0.84	9.73	2.27
750	0.83	9.68	2.32
800	0.90	10.28	1.72
850	0.90	10.20	1.80
900	0.91	10.24	1.76

^1^ Chemical composition is normalized to (Fe + Cr) = 12 for the comparison with SrFe_12-x_Cr_x_O_19_.

## Data Availability

The data presented in this study are available on request from the corresponding author.
